# Scientific Papers by Developmental Biologists in Japan

**DOI:** 10.3390/jdb11010011

**Published:** 2023-03-10

**Authors:** Hideyo Ohuchi, Tsutomu Nohno

**Affiliations:** 1 Department of Cytology and Histology, Okayama University Faculty of Medicine, Dentistry and Pharmaceutical Sciences, 2-5-1 Shikata-cho, Kita-ku, Okayama 700-8558, Japan; 2 Department of Cytology and Histology, Okayama University Medical School, 2-5-1 Shikata-cho, Kita-ku, Okayama 700-8558, Japan; 3 Department of Public Health, Kawasaki Medical School, 577 Matsushima, Kurashiki 701-0192, Japan

We have assembled ten interesting manuscripts submitted by developmental biologists in Japan. We believe that these manuscripts represent a valuable contribution to the specific field of developmental biology. This Special Issue contains six research articles [1,2,3,4,5,6] and four reviews [7,8,9,10].

In the first article [1], Nishiya et al. investigated the period and location of expression of transcription factors in fetal lamb and adult ewe kidneys. They reported the expression patterns in the developing kidney for herbivorous mammals in reference to the omnivorous rodent, showing that WT1 is involved in nephron development, and that Pax2, Pax8, and HNF1β are involved in nephron maturation and collecting duct formation.

Nakajima et al. [2] examined the regeneration polarity of the fin ray during zebrafish caudal fin regeneration. Although the fin rays always regenerated from the proximal margin toward the distal margin, the regeneration-related genes were expressed at both the proximal and distal edges of the hole in the early stage of regeneration, suggesting that the regenerative response occurs at the distal edge. The difference between the proximal and distal margins is a sheet-like tissue that is formed on the apical side of the regenerated tissue at the proximal margin but not at the distal edge. By separating the distal margin from the proximal margin with manipulation, they showed that the sheet-like tissue was formed at the distal margin and that regeneration of the fin ray was also induced. The regenerated fin rays from the distal margin protruded laterally from the caudal fin and then bent distally, and both ends showed the same characteristics as those of the normal fin rays. The authors concluded that fin rays have the ability to regenerate in both directions, although regeneration is restricted to the proximal margin under normal conditions because of preferential formation of the sheet-like tissue on the apical side of the regenerating tissue from the proximal margin.

Goto et al. [3] reported the body axis determination in ascidian early development through the relocalization of maternal determinants, organelles, or unique cell populations in a cytoskeleton-dependent manner. In the ascidian first cell cycle, the myoplasm, including mitochondria, endoplasmic reticulum, and maternal mRNAs move to the future posterior side concomitantly. This translocation consists of first and second phases depending on the actin and microtubule, respectively. To determine the process of transition from the first to the second phase, the authors analyzed the relationship between the cytoskeletons and myoplasmic components during the first cell cycle with an inhibitor. They showed unexpected F-actin accumulation at the vegetal pole during this transition period. The microtubule structure was strongly affected after F-actin depolymerization, and the myoplasmic components were mislocalized, resulting in disordered anteroposterior axis formation. The authors suggest the importance of F-actin during the first cell cycle and the existence of interactions between microfilaments and microtubules, implying the enigmatic mechanisms of ooplasmic segregation.

Kinuhata et al. [4] studied the role of a basic helix-loop-helix (bHLH) transcription factor, Bhlhe40, in chicken eye development, focusing on the stage of optic vesicle morphogenesis, when two retinal domains, the neural retina (NR) and retinal pigment epithelium (RPE), emerge to separate. They reported that *Bhlhe40* is expressed in the prospective and definitive RPE and that its overexpression represses an NR gene, *Vsx2*, while maintaining an RPE gene, *Otx2*. Their attempts to obtain morphological change after expressing dominant negative forms of Bhlhe40 were not successful, possibly because there are many bHLH factors that could dimerize with Bhlhe40 and modify its function. Although it was not demonstrated how the cell behavior in the optic cup was changed after *Bhlhe40* overexpression, this study implicates that *Bhlhe40* is a conserved RPE gene functioning like a node for RPE specification.

Shiraki et al. [5] investigated the role of *wt1* in pronephros development in *Xenopus* embryos, with CRISPR/Cas9 mutation of *wt1*, resulting in reduced expression of pronephros marker genes. WT1 activated transcription of the luciferase reporter gene, and the injection of wild-type or artificially altered transcriptional active *wt1* mRNA disrupted the expression of pronephros marker genes in the embryos. Their results suggest that the appropriate amounts and activity of WT1 protein are required for normal pronephros development in *Xenopus* embryos.

Oishi et al. [6] reported important findings on the role of sonic hedgehog (Shh) in glutamatergic cortical subtype specification. They found that E14.5-born neurons with elevated Shh expression frequently differentiated into layer 4 subtypes as judged by the cell positioning and molecular identity, and that this effect was achieved indirectly through the regulation of cell positioning rather than the direct activation of layer 4 differentiation programs. They provided evidence that Shh as an extrinsic factor plays an important role in the specification of cortical superficial layer subtypes.

Kudo and Ohta [7] discussed the role of an extracellular secreted protein, Akhirin, as a niche molecule during mouse brain formation. Akhirin is structurally related to vitrin and cochlin, and supposedly involved in neural stem cell niche regulation in the central nervous system through heterophilic cell adhesion activity.

Shinozuka and Takada [8] reviewed the most dorsal region of neural tube, the roof plate, as the dorsal organizing center of the developing spinal cord. The dorsal half of the spinal cord is separated by a glial structure called the dorsal median septum. Roof plate cells have been shown to dramatically stretch along the dorsoventral axis with reduction of the spinal cord lumen. During this stretching process, the tips of roof plate cells maintain contact with cells surrounding the shrinking lumen, eventually exposed to the inner surface of the central canal. The authors further discuss the role of Wnt ligands secreted by roof plate cells in morphological changes during spinal cord development.

Funato [9] reviewed DiGeorge syndrome (DGS) and velocardiofacial syndrome (VCFS) caused by a 1.5 to 2.5 Mb hemizygous deletion of chromosome 22q11.2 in humans, focusing on craniofacial anomalies, and summarized the current understanding of the genetic factors that impact DGS/VCFS-related phenotypes. She also reviewed DGS/VCFS mouse models that have been designed to better understand the pathogenic processes of DGS/VCFS.

Yomogita et al. [10] discussed the causes of prolonged post-term delivery in humans in relation to the mouse model, providing a foundation for conducting more systematic research on delayed delivery. The length of the gestation period is controlled not only by the maternal signals, but also by fetal and placental signals. Elucidation of the causes of delayed parturition in humans and animals is essential to understand the mechanisms underlying pregnancy and delivery.

We hope that our readers enjoy these articles, not only for their valuable contributions to the respective fields but also for their introductions to new ideas, new ways of thinking, and experiments on developmental and regenerative processes. We would like to thank all reviewers for their independent evaluations of the submitted articles, and the editorial staff at the *Journal of Developmental Biology* for their efforts in assembling this Special Issue.
